# The view from 10,000 procedures: technical tips and wisdom from master pancreatic surgeons to avoid hemorrhage during pancreaticoduodenectomy

**DOI:** 10.1186/s12893-015-0109-y

**Published:** 2015-11-25

**Authors:** Chad G. Ball, Elijah Dixon, Charles M. Vollmer, Thomas J. Howard

**Affiliations:** Department of Surgery, University of Calgary, Foothills Medical Centre, 1403-29 Street NW, Calgary, Alberta T2N 2T9 Canada; Department of Surgery, University of Pennsylvania, Philadelphia, PA USA; Department of Surgery, Indiana University, Indianapolis, IN USA

**Keywords:** Pancreaticoduodenectomy, Whipple, Hemorrhage, Pancreas

## Abstract

Pancreaticoduodenectomy remains the exclusive technique for surgical resection of cancers located within both the pancreatic head and periampullary region. Amongst peri-procedural complications, hemorrhage is particularly problematic given that allogenic blood transfusions are known to increase the risk of infection, acute lung injury, cancer recurrence and overall 30-day morbidity and mortality rates. Because blood loss can be considered a modifiable factor that reflects surgical technique, rates of perioperative blood loss and transfusion have been advocated as robust quality indicators. We present a correspondence manuscript that outlines peri-procedural concepts detailing a successful pancreaticoduodenectomy with minimal hemorrhage. These tips were collated from master pancreatic surgeons throughout the globe who have performed over 10,000 cumulative pancreaticoduodenectomies. At risk scenarios for hemorrhage include dissections of the superior mesenteric – portal vein, gastroduodenal artery, and retroperitoneal soft tissue margin. General principles in limiting slow continuous hemorrhage that may accumulate into larger total case losses are also discussed. While many of the techniques and tips proposed by master pancreas surgeons are intuitive and straight forward, when taken as a collective they represent a significant contribution to improved outcomes associated with the pancreaticoduodenectomy over the past 100 years.

## Main text

Although the pancreatoduodenectomy is now 100 years of age [1, 2], it remains a formidable procedure with substantial risks. It is also the exclusive technique for surgical resection, and therefore cure, of cancers located within both the pancreatic head and periampullary region. Most modern discussions of peri-procedural complications detail postoperative diagnoses such as pancreatic fistulae, hemorrhage, and delayed gastric emptying [3–5]. This morbidity is not only crucial to patient outcome, but also has a significant impact on a given patient’s ability to obtain adjuvant systemic therapy. Although the overall perioperative morbidity rate has remained relatively consistent over the modern experience at 40 % [6, 7], high volume centers with experienced pancreatic surgeons have substantially improved with regard to successfully treating and temporizing most complications [8, 9]. Despite this robust data, the volume-outcome relationship is inherently complex and continues to be further defined [10–13]. These advances have led to a dramatic reduction in the associated mortality rate from more than 20 % in the 1970s to less than 3 % in recent reports [14–16]. Although improved management of post-operative complications via enhanced antimicrobial therapy, accurate percutaneous catheter drainage and advanced critical care account for a substantial component of this observation, a significant reduction in blood loss during the procedure itself is also postulated to be a dominant contributor.

Two distinct forms of hemorrhage are possible during a pancreatoduodenectomy. These include the spurge of slow ongoing ‘oozing’ during a lengthy procedure, as well as the drama associated with massive hemorrhage, which is typically venous in origin. Hemorrhage is particularly problematic given that allogenic blood transfusions are known to increase the risk of infection, acute lung injury and potentially cancer recurrence via transfusion-induced immunosuppression [17–24]. Blood product transfusions [25–28], in combination with increased duration of the procedure itself [29], have also been shown to substantially increase 30-day morbidity and mortality rates.

Because surgical blood loss can be considered a modifiable factor that reflects surgical technique, rates of perioperative blood transfusion have been advocated as robust quality indicators [24, 29]. Despite this reality, there has been very little discussion of the specific techniques, maneuvers, or surgical outlook that is required to minimize blood loss during pancreatoduodenectomy.

To this end, a small working group was convened to identify current masters of pancreas surgery. These senior surgeons were labeled as international experts after having completed significant volumes of pancreatoduodenectomies. A total of 16 of the 18 targeted surgeons responded with detailed answers. These discussions often involved both email and telephone modalities. Contributing masters included: Hans Beger, Charles Yeo, Murray Brennan, Markus Buechler, Mark Callery, Elijah Dixon, Douglas Evans, David Feliciano, Alan Hemming, Thomas Howard, Bernard Langer, Keith Lillemoe, Henry Pitt, Steven Stasberg, Francis Sutherland, and Bryce Taylor. These masters have a cumulative reported experience of 10,420 pancreatoduodenectomy procedures. This project was approved by the Institutional Review Board at the University of Calgary. No patient data was utilized. No data repository was therefore required. No patient participants were included (surgeon opinions of technique and theory only), therefore no patient/participant consent was utilized.

## Scenarios ‘at risk’ for hemorrhage

### Superior mesenteric – portal vein

Mobilization of the superior mesenteric – portal vein (SMV-PV) confluence is clearly a scenario with substantial risk for hemorrhage. These venous structures are thin walled, and particularly friable in the context of glandular inflammation (acute), ongoing scarring (chronic pancreatitis), and/or mechanical distortion (locally advanced tumors). To minimize this risk, an early and complete Kocherization maneuver is critical for exposure and visualization, as well as for potential left-handed gentle compression in the setting of hemorrhage (i.e. squeezing the pancreatic head and SMV-PV confluence against the surgeon’s own thumb). The Kocher maneuver should occur shortly after the initial mobilization of the right colon and transverse mesocolon.

Pre-emptive control of major veins can also be essential in all but the most straightforward surgical fields. Techniques include careful dissection and control of the superior mesenteric vein (SMV) and portal vein (PV) with vessel loops. Dissecting the SMV inferiorly to ensure exposure of a reasonable length of ‘clampable’ vessel is very helpful. One must also avoid narrow tunnels in poor or difficult conditions. This principle can be achieved by elevating the inferior border of the pancreas to widen the exposure/approach to the retropancreatic tunnel. The same technique can be performed on the superior border of the pancreas to ensure finger access and potential occlusion of the splenic vein to the left of the SMV-PV confluence. There should be no rush to create the retropancreatic tunnel anterior to the PV in the cadence of a normal pancreatoduodenectomy procedure. In fact, numerous master surgeons routinely perform this step last (i.e. after dissection of the SMV below, PV above, common hepatic artery above, and looping of the common bile duct), so that if a SMV-PV injury does occur, it is significantly easier to address on a rapid basis.

If a tear occurs in the portal vein early in the procedure, rapid transection of the pancreatic neck while preparing for suture ligation of a potentially bleeding splenic vein is helpful. Ideally, this can be done immediately to the left of the portal vein. It should be noted that the splenic and/or inferior mesenteric veins may be ligated with impunity in patients with ongoing massive hemorrhage [30]. If standard maneuvers to arrest venous hemorrhage fail (i.e. packing the retropancreatic tunnel with topical hemostatics and applying pressure), immediate transection of the stomach/duodenum can also be immensely helpful by increasing direct visualization and exposure. Long Allis clamps are particularly useful for venous hemorrhage in any location. These clamps can be applied directly to the venous laceration, with potential conversion to a formal vascular clamp once the hemorrhage has been controlled. It should also be noted that although portal vein ligation in the context of penetrating trauma has been shown to be associated with improved outcomes over attempts at repair [31], patients undergoing pancreatoduodenectomy are generally not candidates for ligation without reconstruction given (1) the associated portal dissection, (2) lack of collaterals, and (3) generally poor physiologic reserve.

An increasingly common scenario involves hemorrhage of colic veins tearing from their insertion into the SMV-PV complex due to overzealous retraction of large and heavy transverse colons in obese patients. Clearly, obesity creates more difficult technical demands, with or without associated hemorrhage. Finally, in catastrophic hemorrhage requiring prolonged clamping of the SMV, massive intestinal congestion and swelling may be improved by intermittent occlusion of the SMA. This maneuver should be reserved for only the most precarious of scenarios.

### Gastroduodenal artery

Dividing the gastroduodenal artery (GDA) in scenarios with large adjacent tumors can also be particularly treacherous. The goal to leave a reasonably generous length of GDA stump as a target zone for potential angioembolization of future GDA hemorrhage (autologous clot or coils), is also challenging in the context of bulky tumors. As a result, if the GDA is short and friable, avoid tying the vessel in continuity. It may be more elegant to gently clamp the common hepatic artery proximally and then transect the GDA with a sharp scalpel in preparation for subsequent fine suture ligation. Although this technique results in a small amount of blood loss, the GDA stump typically remains in good condition. It is clear that any carelessness around the GDA leads to an increased risk of pseudoaneurysms, and subsequent post-pancreatectomy hemorrhage. In cases of propagating an intimal fracture/plaque from the GDA into the common hepatic artery, acute occlusion of the common hepatic artery must be considered and addressed. It must also be remembered that if the surgeon encounters atypical arterial anatomy (i.e. not previously noted on the preoperative cross-sectional imaging), arresting dissection and detailing arterial delineation with doppler ultrasound is crucial.

### Retroperitoneal soft tissue dissection

Dissection of the retroperitoneal margin is a step of particular risk. Wide and complete mobilization of the portal vein off of the pancreatic head and uncinate is vital. This wide exposure allows identification and ligation of the ubiquitous first lateral branch of the PV at the top of the pancreatic head, as well as any aberrant branches along its entire length. This pre-emptive maneuver also provides complete identification of the first jejunal PV branch at the bottom of the dissection for control and ligation if needed. This anatomy is generally predictable and must be respected with accurate dissection.

Lateral retraction of the pancreatic head complex in combination to medial retraction of a well-mobilized portal vein is also important. Furthermore, lateral retraction of the SMV-PV itself allows direct SMA branch control. While close dissection along the SMA is particularly crucial with regard to obtaining a negative oncologic margin, one must always be aware of creating intimal SMA flaps or injury with rough technique. SMA intimal flap creation, as well as ongoing hemorrhage, is also possible without careful ligation (and subsequent fracturing) of the inferior pancreaticoduodenal artery, or other arterial branches from the SMA into the head of the pancreas. This is a particularly important issue in patients with severe fibrosis of the retroperitoneum. It also applies to ligation of the posterior-superior branch of the pancreatoduodenal artery. As a general principle however, direct visualization and palpation of the SMA will lead to safer surgery, and hopefully better oncologic margins. In summary, complete dissection of the SMV-PV confluence with lateral retraction will allow the surgeon to elevate the entire retroperitoneum out of the wound, and provide adequate digital compression and control of any hemorrhaging branches from the SMA using a thumb. If significant bleeding occurs during transection of the retroperitoneal pancreatic margin, then the pancreatic complex should be excised as quickly as possible prior to arresting hemorrhage (assuming all remaining components of the procedure are already complete). This may be performed with a non-cutting stapler, long and narrow clamps, or cautery. Clearly, reconstruction must wait until immaculate hemostasis has been achieved.

## General principles

Preoperative planning and high fidelity cross sectional imaging are crucial to the bloodless success of any pancreatoduodenectomy. This allows the surgeon to predict where the vast majority of problems will occur, and as a result, prepare well ahead of time with many of the techniques and tricks listed above. Do not move on to the next step in a pancreatoduodenectomy until the operative field is dry. If you believe dissection of the PV is high risk in the setting of a planned PV resection, harvest your reconstruction conduit (if autologous) prior to dividing the pancreatic neck and uncinate margin.

Regardless of the specific location, upon encountering significant ongoing hemorrhage, remember to: (1) stop and apply pressure, (2) maintain your composure and alert anesthesia and the nursing staff of significant hemorrhage, (3) ‘work the problem’ and contemplate your options, (4) call for senior assistance, (5) obtain a second sucker and open all vascular instruments, (6) arrest the hemorrhage prior to attempting a repair, and (7) do not flail (i.e. don’t repeat the same maneuvers to arrest hemorrhage if they do not work the first time).

## Minimally invasive methodologies

Two dominant minimally invasive methods for pancreaticoduodenectomy deserve mention. More specifically, completely laparoscopic or robotic and/or hybrid combinations of these two techniques are increasingly common in some institutions. While debate continues to rage with regard to the efficacy, utility and true benefit of these approaches, the principles of thoughtful preoperative assessment, avoiding initial hemorrhage, as well as temporizing and subsequently arresting bleeding discussed above remain applicable to minimally invasive procedures. The 2 obvious caveats to these concepts surround (1) the inability of the surgeon to apply left handed pressure/occlusion to arrest hemorrhage, and (2) the time interval that is associated with converting to an open procedure in the context of ongoing hemorrhage. Rapidly inserting additional 5 mm ports through which compressive instruments may be placed can be extremely helpful. Furthermore, in the case of venous hemorrhage, increasing the intraperitoneal insufflation pressure can also assist in temporizing bleeding (+/- allow time for conversion). Regardless of the specific source of hemorrhage and level of surgical experience however, some form of temporary control must generally be achieved prior to converting to a fully open procedure. This will reduce the volume of interim hemorrhage and allow a more controlled conversion. Similarly, numerous temporary topical hemostatic agents may also be helpful in conjunction with instrument pressure. These include, but are not limited to liquid and foam based agents, as well as solid and semi-solid formats.

## Conclusions

While many of the techniques and tips proposed by master pancreas surgeons are intuitive and straight forward, when taken as a collective they represent a significant contribution to improved outcomes associated with the pancreatoduodenectomy over the past 100 years (Table 1). It is also clear that attention to detail during the resection, with regard to the avoidance of *all* blood loss (both low volume ‘oozing’ and massive hemorrhage), is paramount to avoiding the subsequent need for blood product transfusion. As a result, minimizing hemorrhage via these techniques is clearly associated with quality outcomes and indicators.

**Table 1:** Avoiding hemorrhage during pancreaticoduodenectomy checklist

Tips:
1. Complete preoperative cross-sectional imaging is essential
2. Thoughtful preoperative resectional planning avoids hemorrhage
3. Do not proceed to the next step until the current one is hemostatic
4. Left handed compression in the context of a complete Kocherization is crucial
5. Widen the approach/exposure to the SMV/PV at the borders of the pancreas
6. Allis clamps never met veins they didn’t like
7. Leave the GDA stump as long as is technically possible
8. Direct palpation and visualization of the SMA minimizes the risk of injury
9. Harvest reconstruction conduit prior to the vascular resection
10. Stay calm and obtain experienced assistance when hemorrhage is significant
11. Possess a progressive hierarchy of techniques to arrest haemorrhage
12. Principles are consistent for both open and minimally invasive approaches

## Interesting quotes from the masters

“Am I the only one who finds anterior branches to the SMV-PV?”

“Do the hard parts of the resection last”

“Spend as much time in the operating theater with your partners as you can…check all egos at the door”

“Don’t turn an affair of the mind into an affair of the heart by trying to remove a tumor that is unresectable”

“Always enlarge the incision…exposure is half of the battle in ongoing hemorrhage”

“The Allis clamp never met a vein it didn’t like”

“Any surgeon engaging in a Whipple, must be facile with vascular surgical techniques”

“Sometimes small sutures with big bleeding lead to tears and further bleeding, so if you’re really in trouble, sew vessels shut and reconstruct later”

“When I’m asked “How do you know when to turn back?”…I answer “it’s very easy; always about 7 s after I should have””

“Although important, technique can not defeat biology”

“Slow the #@ down…this is not liver surgery!”

## Abbreviations

GDA: Gastroduodenal artery
PV: Portal vein
SMA: Superior mesenteric artery
SMV: Superior mesenteric vein
